# Parental and Child Self‐Efficacy Explaining Food Intake through Self‐Regulation: A Dyadic Prospective Study

**DOI:** 10.1111/aphw.12225

**Published:** 2020-09-13

**Authors:** Karolina Zarychta, Anna Banik, Ewa Kulis, Karolina Lobczowska

**Affiliations:** ^1^ SWPS University of Social Sciences and Humanities in Wroclaw Wroclaw Poland

**Keywords:** child‐parent dyads, energy‐dense food intake, fruit and vegetable intake, self‐efficacy, self‐regulation

## Abstract

**Background:**

According to social cognitive theory and socio‐ecological models, self‐efficacy and temptation‐related self‐regulation (the use of distraction or suppression) are modifiable predictors of health behaviors, such as food intake. Yet, there is limited evidence explaining how these factors are interlinked among parent‐child dyads. This study investigated indirect effects of parental and child self‐efficacy on food intake, via parental and child self‐regulation.

**Methods:**

The prospective study (the baseline [T1] and the 10‐month follow‐up [T2]) enrolled 924 parent‐child dyads (1,848 individuals; 54.3% girls, aged 5–11 years, 88.9% mothers). Dyads were interviewed or completed self‐report measures. Path analyses with maximum likelihood estimation were conducted.

**Results:**

Child self‐efficacy and distraction (T1) mediated between parental self‐efficacy (T1) and higher levels of child fruit and vegetable intake (T2). No significant mediating effects of suppression were found, nor indirect effects of parental self‐efficacy (T1) on energy‐dense food intake (T2).

**Conclusion:**

Health promotion interventions aiming at changing fruit and vegetable intake among 5–11‐year‐old children should target enhancing parental and child self‐efficacy that may facilitate the use of self‐regulation and, in turn, healthy diet.

**Abbreviations**
BMI: body mass index
T: time
H: hypothesis

## INTRODUCTION

The World Health Organization (WHO, 2018) guidelines for nutrition among adults and children recommend consuming at least 400 grams of fruit and vegetable daily, and consuming no more than 30 per cent of total calorie intake from energy‐dense food (high in calories and saturated fatty acids). In children, high fruit and vegetable intake and low levels of energy‐dense food intake may have long‐term health benefits such as reducing the risk of becoming overweight or obese, as well as developing noncommunicable diseases (NCDs) in adulthood, such as cardiovascular diseases, cancer, and diabetes (Stanaway et al., 2018). In adults, healthy nutrition behavior may decrease the risk of NCDs and excessive body mass or help to reduce symptoms of NCDs that are already present (Stanaway et al., 2018). Yet, the percentage of people meeting such recommendations remains low (WHO, 2018) and, in consequence, a diet low in fruit and vegetables and high intake of energy‐dense food is linked to increased mortality rates (Stanaway et al., 2018).

Social cognitive theory (SCT; Bandura, 1997) is a widely used model that explains behavior change with the continuous interaction of personal and environmental factors, including interactions between parents and children. The influence of parent–child interactions on healthy diet is strongest when children are aged 5–11 years old (Cislak et al., 2012). Previous research indicated that children as young as 5–9 years old perceive their parents as the primary source of nutrition information (Brindal, Hendrie, Thompson, & Blunden, 2012). During the adolescence period, parental influence decreases while peers and media start to play a significant role in shaping adolescents’ beliefs and behaviors (Cislak et al., 2012). As indicated in socio‐ecological models (Davison & Birch, 2001; Townsend & Foster, 2011), food intake among children is explained by the interaction of a variety of personal and environmental factors (Townsend & Foster, 2011). For example, parental beliefs and behaviors are assumed to form the main determinants of child’s beliefs and food intake (Davison & Birch, 2001; Savage et al., 2007). More specifically, the examples of such beliefs and behaviors can be parental and child self‐efficacy for fruit and vegetable intake, and temptation‐related self‐regulation to deal with energy‐dense food.

Self‐efficacy is one of the main concepts in SCT, and can be defined as people’s beliefs in their own capabilities to perform or achieve a behavior or a goal, which determine how individuals think, feel, and behave (Bandura, 1997). According to SCT, self‐efficacy is central to behavior change since people are more likely to perform a behavior when they are confident of their abilities to do it. High levels of self‐efficacy for fruit and vegetable intake and high levels of self‐efficacy for energy‐dense food intake reduction are associated with high levels of fruit and vegetable intake in adults (Guillaumie et al., 2012) and 6–12‐year‐old children (Baranowski et al., 2010; Gaines & Turner, 2009). The two constructs are also related to low energy‐dense food intake (e.g. Churchill et al., 2018; Guillaumie et al., 2012). High fruit and vegetable intake and low energy‐dense food intake are indicators of a healthy diet (WHO, 2018). Therefore, it seems appropriate to include self‐efficacy for fruit and vegetable intake or self‐efficacy for energy‐dense food intake reduction in research explaining food intake in families with young children. Moreover, as shown in twin studies (Cai & Luo, 2017), child self‐efficacy is explained by heritability and, to a lesser extent, by environmental factors (e.g. parental modeling). Thus, parental self‐efficacy may be assumed to precede children’s self‐efficacy and influence its formation. Previous studies have indicated that self‐efficacy and self‐regulation operate in concert when predicting health outcomes and are listed among the most significant determinants of health (Anderson et al., 2007).

Self‐regulation can be defined as deliberate attempts to self‐control one’s behavior, such as food intake (Herman & Polivy, 2004). Overall, individuals with high self‐regulation abilities report higher fruit and vegetable intake than individuals with lower self‐regulation abilities; such associations were confirmed among adults (Hagger et al., 2019; Stadler et al., 2010) and 9–14‐year‐old children (Gaines & Turner, 2009; Wills et al., 2007). Self‐regulation referring to food intake may be represented as different attempts (both cognitive and behavioral) to refrain from energy‐dense food while being tempted by it (De Vet et al., 2014). Several such self‐regulation strategies are described in the existing literature, including strategies directly addressing temptations (avoidance of temptations and controlling temptations), strategies directly addressing the behavioral goal (goal and rule setting, and goal deliberation), and strategies addressing the meaning of temptations (suppression and distraction) (De Vet et al., 2014). Yet, in the case of children, the first two groups of self‐regulation strategies seem to be more parent‐dependent. Parents are considered to be the primary source of children’s food choices (Brindal, Hendrie, Thompson, & Blunden, 2012; Luszczynska et al., 2013), and children may not be able to avoid or control energy‐dense food intake or follow the goal to eat more fruit and vegetables when the food product provision depends only (or mainly) on parents. In contrast, the self‐regulation strategies addressing the meaning of temptations (suppression and distraction) may be used by parents and children, regardless of parental food product provision. Therefore, the present study will focus on self‐regulation addressing the meaning of temptation. In the present study suppression is defined as consciously trying to reduce the impact of tempting food cues on one’s behavior, and distraction as shifting one’s attention from a tempting food cue to another object (De Vet et al., 2014). Suppression and distraction were found to be associated with high fruit and vegetable intake and low energy‐dense food intake (De Vet et al., 2014).

According to the Health Action Process Approach model (HAPA; Schwarzer, 2008), health behavior change involves the initial intention phase in which individuals’ beliefs such as self‐efficacy play a crucial role, followed by the behavior maintenance phase. Also, SCT suggests that self‐efficacy is an important determinant of the maintenance of a behavior (Bandura, 1997). In the behavior maintenance phase, self‐regulatory actions are the most important (e.g. planning or action control in the HAPA; Schwarzer, 2008). Self‐regulation is suggested to be predicted by self‐efficacy (Rothman et al., 2004; Schwarzer, 2008). Therefore, it is more likely that self‐regulation would mediate the association between self‐efficacy and food intake (e.g. Anderson et al., 2007) than the other way round (e.g. Annesi, 2011).

Existing empirical evidence suggests that parental self‐efficacy and self‐regulation are linked to child food intake. For example, several cross‐sectional studies have confirmed positive associations between parental self‐efficacy and parental reports of high fruit and vegetable intake among children (Kokolaki et al., 2018; Norman et al., 2017; Parekh et al., 2017) and low energy‐dense food intake among children (Norman et al., 2017; Parekh et al., 2017). Moreover, targeting parental self‐efficacy or parental self‐regulation (Gholami et al., 2014; Hodder et al., 2019) in interventions promoting healthy food intake among children was found to contribute to high fruit and vegetable intake, and low energy‐dense food intake. Parents are assumed to be one of the most important health promotion mediums for their children, and are often the main target of child obesity prevention programs (Beydoun & Wang, 2009). On the other hand, research also showed that parental beliefs and behaviors are only one of many different factors explaining child nutrition; for example, children’s food intake only moderately resembles the food intake of their parents (Beydoun & Wang, 2009). Therefore, it seems important to investigate not only parental beliefs and behaviors, but also those of their children. Yet, we found no research testing the indirect dyadic associations between parental and child self‐efficacy, parental and child self‐regulation strategies, and parental and child food intake. This study aims at filling that void.

Self‐efficacy and self‐regulation strategies are well‐known modifiable predictors of food intake (Gestsdottir & Lerner, 2008). Operating in concert, self‐efficacy and self‐regulation were found to explain together as much as 17 per cent variance of fruit and vegetable intake (Annesi, 2011). Thus, it is important to understand the relationships between these variables in the family context to design more effective interventions targeting healthy nutrition among both children and parents (Townsend & Foster, 2011). Unfortunately, the majority of the previous studies have applied cross‐sectional designs and focused primarily on direct and bivariate associations between the parental self‐efficacy and child food intake (Kokolaki et al., 2018) or between parental self‐regulation strategies and child food intake (Gholami et al., 2014). Only one prospective study (Anderson et al., 2007) assessed the indirect associations between self‐efficacy and food intake via self‐regulation strategies. However, it involved a short follow‐up period (3 months) and enrolled only adults reporting their data on their own beliefs and behaviors. Moreover, most of the previous studies employed parental self‐reports only (Gholami et al., 2014; Kokolaki et al., 2018; Norman et al., 2017), therefore, they were testing associations between parental perceptions of their own behaviors and parental perceptions of their children’s behaviors. In conclusion, there is a need for further research testing the indirect effects of parental and child self‐efficacy, parental and child self‐regulation, and parental and child food intake.

To address the limitations of current evidence, a dyadic prospective study was conducted. In particular, two models were created to test four hypotheses.

Model 1:

*Hypothesis 1* (*H1*): The indirect effect of higher parental self‐efficacy (measured at Time 1 [T1]) on higher child fruit and vegetable intake (measured at Time 2 [T2]) would be mediated by higher child self‐efficacy (T1), higher levels of parental suppression (T1), and higher levels of child suppression (T1).
*Hypothesis 2* (*H2*): The indirect effect of lower parental self‐efficacy (T1) on lower child energy‐dense food intake (T2) would be mediated by lower child self‐efficacy (T1), lower levels of parental suppression (T1), and lower levels of child suppression (T1).

Model 2:

*Hypothesis 3* (*H3*): The indirect effect of higher parental self‐efficacy (T1) on higher child fruit and vegetable intake (T2) would be mediated by higher child self‐efficacy (T1), higher levels of parental distraction (T1), and higher levels of child distraction (T1).
*Hypothesis 4* (*H4*): The indirect effect of lower parental self‐efficacy (T1) on lower child energy‐dense food intake (T2) would be mediated by lower child self‐efficacy (T1), lower levels of parental distraction (T1), and lower levels of child distraction (T1).

## METHODS

### Participants

Parent‐child dyads were invited to participate in the study. Specifically, parents (98.6%) or legal guardians (1.4%; henceforth called “parents”) of children aged 5–11 years old without physical impairments resulting in major movement restrictions (e.g. cerebral palsy) were included in the study. Parents were the main caregivers in terms of preparing food and time spent with a child. Data were collected as a part of a larger study testing parental and child psychosocial determinants of body mass (Liszewska et al., 2018; Zarychta et al., 2019).

At Time 1 (T1, the baseline), 924 dyads participated (1,848 individuals), and 571 dyads (1,142 individuals) participated at Time 2 (T2, the 10‐month follow‐up). Of the parents (*N* = 924) participating at T1, 88.9 per cent were women and 11.1 per cent were men aged between 23 and 66 years old (*M* = 36.27, *SD* = 5.52). Their body mass index (BMI) ranged from 16.53 to 46.88 (*M* = 24.57, *SD* = 4.47) with the majority of them (59.2%) having normal body weight, 26.4 per cent being overweight, 12.0 per cent having obesity, and 2.4 per cent being underweight. At T2, adult participants (*N* = 571) were women (91.6%) and men (8.4%) between 23 and 67 years of age (*M* = 36.27, *SD* = 5.51). At T1, child participants (*N* = 924) aged 5–11 years old (*M* = 8.23, *SD* = 1.41) were girls (54.3%) and boys (45.7%) with a BMI range of 11.24 to 33.74 (*M* = 17.07, *SD* = 2.98). The majority of children had normal body weight (66.8%), 18.2 per cent were overweight, 5.8 per cent were obese, and 9.2 per cent were underweight (according to the International Obesity Task Force [IOTF] cut‐off points adjusted for age and gender; Cole & Lobstein, 2012). Children (*N* = 571) participating at T2 were girls (57%) and boys (43%) aged between 6–12 years old (*M* = 8.81, *SD* = 1.40). All participants were Caucasian.

### Procedure

Data were collected from 2011 through 2015 in 26 villages, towns, and cities of six administrative regions of Poland representing different levels of the mean household income. Participants were provided with information about the research aims and procedure. Afterwards, informed consent from parents (about their own and child participation) and assent from children were collected, and de‐identified codes were assigned to ensure participants’ anonymity across the measurement points. Parents completed the questionnaires in a room separately from their children. Younger children (aged 5–8 years old) were interviewed using a structured interview schedule while older children (aged 9–11 years old) completed a questionnaire at school, in general practitioners’ offices, or at participants’ homes.

At T1, parents and children provided their data referring to their self‐efficacy, self‐regulation strategies (namely, suppression and distraction), fruit and vegetable intake, and energy‐dense food intake. At T2, participants reported their data on fruit and vegetable intake and energy‐dense food intake. Participants’ body weight and height were measured with certified scales and rods at both T1 and T2. During the follow‐up, study personnel revisited the study sites after contacting parents by phone to repeat the measurements. The time interval between the measurement points was chosen to limit longitudinal dropout since it comprises one school year (from the beginning of the school year, T1; to its end, T2).

The study was approved by the Internal Review Board at SWPS University of Social Sciences and Humanities, Wroclaw, Poland. All procedures were in accordance with the ethical standards of the institutional research ethics committee and in line with the 1964 Helsinki Declaration and its later amendments.

### Materials

As the differences in the assessment format may have influenced the associations between parent and child variables, the same measures were applied to both members of the dyad (Kenny et al., 2006). The feasibility of item‐wording for children was tested in a pilot study with *n* = 18 children (aged 5–11 years old). Children were asked to explain the instructions and the items in their own words and to indicate any phrases they did not understand/were unsure of. The pilot study showed that the children were able to correctly classify their behaviors referring to self‐efficacy, self‐regulation, and food intake.

*Self‐Efficacy (T1)*. Parental and child self‐efficacy for fruit and vegetable intake (for brevity: self‐efficacy) was measured with four items each, based on Baranowski et al. (2010), for example: “I am confident that I can eat three portions of fruit a day”, and “I am confident that I can eat three portions of vegetables a day”. The portion was defined as the amount fitting into a cupped hand. The responses ranged from 1 (“definitely not”) to 4 (“definitely yes”). All items were summed to give the total score for self‐efficacy. Higher total scores represent higher self‐efficacy. Average level of self‐efficacy among parents was *M* = 12.25, *SD* = 2.85, and *M* = 10.48, *SD* = 3.03 among children.

*Suppression and Distraction (T1)*. The two temptation‐related self‐regulation strategies, suppression and distraction, were measured among parents and children with four items each from the Tempest Self‐Regulation Questionnaire for Eating (TESQ‐E; De Vet et al., 2014), for example: “If I go to a party with lots of snacks, I ignore the food” (for suppression), “If I feel like eating something, I call a friend instead” (for distraction). The responses ranged from 1 (“definitely not”) to 4 (“definitely yes”). Respective items were summed to give the total score for suppression and distraction. Higher total scores represent higher levels of suppression or distraction. Average level of suppression among parents was *M* = 8.83, *SD* = 2.29, and *M* = 8.43, *SD* = 2.53 among children. Average level of distraction among parents was *M* = 8.22, *SD* = 2.34, and *M* = 8.87, *SD* = 2.82 among children.

*Fruit and Vegetable Intake (T1 and T2)*. Parental and child fruit and vegetable intake were measured with two items each, based on Lally et al. (2011): “How often did you eat a portion of fresh fruit in the last two weeks?” and “How often did you eat a portion of vegetables in the last two weeks (fresh, boiled or fried without fat)?” A portion was defined as the amount fitting into a cupped hand. The responses were given on a 6‐point scale: 1 (“once a week or less”), 2 (“almost every day of the week”), 3 (“once a day”), 4 (“twice a day”), 5 (“three times a day”), 6 (“four or more times a day”). Both items were summed to give the total score for fruit and vegetable intake. Higher total scores represent higher intake of fruit and vegetables. Average level of fruit and vegetable intake among parents was *M* = 5.06, *SD* = 1.79 at T1 and *M* = 5.14, *SD* = 1.52 at T2. Average level of fruit and vegetable intake among children was *M* = 5.65, *SD* = 2.07 at T1 and *M* = 5.57, *SD* = 1.65 at T2.

*Energy‐Dense Food Intake (T1 and T2)*. Parental and child energy‐dense food intake were measured with two items each, based on Lally et al. (2011): “How often did you eat fatty food (e.g. pizza, chips, food with dressings) in the last two weeks?” and “How often did you eat sweets (e.g. chocolate bars, wafers, cakes) in the last two weeks?” The responses were given on a 6‐point scale: 1 (“once a week or less”), 2 (“almost every day of the week”), 3 (“once a day”), 4 (“twice a day”), 5 (“three times a day”), 6 (“four or more times a day”). Both items were summed to give the total score for energy‐dense food intake. Higher total scores represent higher intake of energy‐dense food. Average level of energy‐dense food intake among parents was *M* = 3.43, *SD* = 1.65 at T1 and *M* = 3.39, *SD* = 1.40 at T2. Average level of energy‐dense food intake among children was *M* = 4.55, *SD* = 2.08 at T1 and *M* = 4.34, *SD* = 1.63 at T2.

*Body Weight and Height (T1 and T2)*. Parental and child body weight and height were assessed with standard medically approved telescopic height measuring rods and floor scales (scale type: BF‐100 or BF‐25; Beurer, Germany, measurement error < 5%). For parents, BMI was calculated using body weight and height: BMI = weight (kg)/height^2^ (m^2^). For children, age‐ and gender‐specific BMI *z*‐score values were calculated with the WHO AnthroPlus macro (WHO, 2011). Parental BMI and child BMI *z*‐scores were accounted for as covariates in the analyses.

### Data Analysis

G*Power calculator (Faul et al., 2007) was used to determine the sample size. Assuming small effect sizes (*f* ^2^ = 0.03) and accounting for potential confounders (listed below), the sample size was estimated for at least 1,800 individuals (i.e. 900 dyads).

Analyses were performed with SPSS version 25 and IBM AMOS 25. Path analyses with maximum likelihood estimation were conducted (Byrne, 2010). A total of 1.8 per cent of the complete data were missing. The total attrition rate was 38.2 per cent. Data missing at T1 or due to longitudinal dropout at T2 were accounted for with the full information maximum likelihood (FIML) (Byrne, 2010). Little’s MCAR test indicated that the missing data patterns were random, Little’s *χ^2^*(1405) = 201.34, *p* = .928. Mardia’s coefficient of multivariate normality indicated moderate non‐normality (48.77 for the hypothesised model).

Several model‐data fit indices were applied. A cut‐off point of ≤ .08 for the root mean square error of approximation (RMSEA), and ≤ .09 for the standardised root mean square (SRMR) were used (Byrne, 2010) as well as a cut‐off point of ≥ .90 for the comparative fit index (CFI), Tucker‐Lewis index (TLI), the goodness‐of‐fit index (GFI), and the normed fit index (NFI) (Byrne, 2010). The indirect effects were evaluated with their unstandardised effect coefficients, after applying 10,000 bootstraps (95% confidence intervals).

Highly correlated mediators may result in multicollinearity affecting the estimation of their partial associations with the dependent variable (Hayes, 2013). Therefore, since the associations between both parental and child self‐regulation mediators were high over the two measurement points, it was decided to run two models to test the hypotheses (each model testing the mediating role of a different self‐regulation strategy). The first of two hypothesised models assumed that the independent variable (IV: parental self‐efficacy at T1) would be associated with three mediators: first, with child self‐efficacy (T1), which would be sequentially related to two parallel mediators (parental suppression [T1] and child suppression [T1]). In turn, the mediators were assumed to predict four dependent variables (DV): parental fruit and vegetable intake (T2), parental energy‐dense food intake (T2), child fruit and vegetable intake (T2), and child energy‐dense food intake (T2). The second of the two hypothesised models assumed that parental self‐efficacy (IV; T1) would be associated with three mediators: first, with child self‐efficacy (T1), which would be sequentially related to two parallel mediators (parental distraction [T1] and child distraction [T1]) predicting four DVs (parental and child food intake; T2). Ideally, each mediator should be measured at a different time point to establish temporal precedence (MacKinnon, 2008). As the present study used only two measurement points, it was decided to measure both the IV and the mediators (child self‐efficacy, child and parental self‐regulation strategies) at T1, and the DV at T2. In line with theoretical models (e.g. Davison & Birch, 2001), variables used as mediators in our study are assumed to be the determinants of health behaviors and precede health behaviors. The determinants are usually measured at earlier time points than DV (for a similar approach see, e.g. Zarychta et al., 2016).

Analyses for the hypothesised models were conducted. The following covariates were accounted for: parental and child gender, parental and child age (T1), parental and child fruit and vegetable intake (T1), parental and child energy‐dense food intake (T1), parental BMI (T1 and T2), and child BMI *z*‐score (T1 and T2). All parental and child variables were assumed to covary. If the indirect effect of the parental self‐efficacy on any of the child food intake variables were to occur in any of the two models, it may be statistically significant even if one of the component paths is not significant.

## RESULTS

### Preliminary Analysis

Parents who participated at both T1 and T2 measurements did not differ from dropouts in terms of self‐efficacy, suppression, distraction, fruit and vegetable intake, energy‐dense food intake, BMI, all *F*s < 2.25, *p*s > .134, or gender, *χ* ^2^(1) = 0.94, *p* = .332. Children who participated at both measurements did not differ from dropouts in terms of suppression, distraction, fruit and vegetable intake, energy‐dense food intake, BMI *z*‐score, all *F*s < 2.65, *p*s > .133, or gender, *χ* ^2^(1) = 0.69, *p* = .405. Child dropouts and child completers differed in terms of self‐efficacy, *F*(1, 878) = 8.29, *p* = .004, with child dropouts being less self‐efficacious (*M* = 10.27, *SD* = 3.23) than completers (*M* = 10.58, *SD* = 2.85, Cohen's *d* = 0.10, 95% CI [−0.30, 0.09]). They also differed in terms of age, *F*(1, 878) = 19.46, *p* < .001, with child dropouts being older (*M* = 8.52, *SD* = 1.51) than completers (*M* = 8.44, *SD* = 1.26, Cohen's *d* = 0.06, 95% CI [−0.03, 0.15]).

Parental fruit and vegetable intake, and parental energy‐dense food intake remained unchanged between T1 and T2 (fruit and vegetable: *t*(1, 923) = −1.84, *p* = .066, energy‐dense food: *t*(1, 923) = 1.27, *p* = .206). In the case of children, child fruit and vegetable intake remained unchanged between T1 and T2, *t*(1, 923) = 1.43, *p* = .152. However, child energy‐dense food intake at T1 (*M* = 4.55, *SD* = 2.08) was significantly higher than at T2 (*M* = 4.34, *SD* = 1.63), *t*(1, 923) = 3.41, *p* = .001, Cohen's *d* = 0.11, 95% CI [0.01, 0.21].

Means, standard deviations, reliability coefficients for all the study’s measures as well as bivariate correlations between study variables (for the total sample of *N* = 924 dyads; *N* = 1,848 individuals) are presented in Table 1.

**TABLE 1:** Descriptive Statistics, Reliability, and Correlations between the Study Variables (*N* = 924 parent‐child dyads)

		*M (SD)*	*α*	*2*	*3*	*4*	*5*	*6*	*7*	*8*	*9*	*10*	*11*	*12*	*13*	*14*	*15*	*16*	*17*	*18*	*19*	*20*	*21*	*22*
1	Self‐efficacy (P, T1)	12.25 (2.85)	.87	.21 ***	.12 ***	.04	.10 **	.06	.35 ***	.34 ***	.15 ***	.17 ***	−.15 ***	−.13 ***	−.01	−.02	−.03	−.03	.03	< .01	.09 **	.03	.01	−.01
2	Self‐efficacy (Ch, T1)	10.48 (3.03)	.82		.05	.16 ***	−.03	.14 ***	.16 ***	.14 ***	.37 ***	.32 ***	−.09 **	−.06	−.05	−.05	−.01	−.02	.03	.01	.08	.16 ***	< .01	< .01
3	Suppression (P, T1)	8.83 (2.29)	.67			.12 ***	.64 ***	.09 **	.17 ***	.14 ***	.03	.05	−.21 ***	−.24 ***	−.07 *	−.06	.06	.06	.09 **	.04	< .01	−.06	.14 ***	.01
4	Suppression (Ch, T1)	8.43 (2.53)	.59				0.09 **	.57 ***	.05	.03	.20 ***	.16 ***	−.05	−.06	−.01	−.02	.04	.04	.05	< .01	.06	.12 ***	−.02	< .01
5	Distraction (P, T1)	8.22 (2.34)	.74					.11 ***	.13 ***	.15 ***	< .01	.04	−.15 ***	−.14 ***	−.10 **	−.06	.11 ***	.12 ***	.11 ***	.04	−.01	−.11 ***	.15 ***	< .01
6	Distraction (Ch, T1)	8.87 (2.82)	.69						.03	−.03	.22 ***	.18 ***	−.06	−.01	.02	< .01	.06	.07 *	.13 ***	.06	.07 *	.12 ***	−.03	.03
7	Fruit and vegetable intake (P, T1)	5.06 (1.79)								.68 ***	.18 ***	.26 ***	< .01	−.04	−.02	−.05	−.10 **	−.09 **	.05	.06	< .01	.02	.13 ***	.04
8	Fruit and vegetable intake (P, T2)	5.14 (1.52)									.14 ***	.31 ***	−.03	.02	−.01	.01	−.07 *	−.07 *	< .01	.03	.03	.01	.15 ***	.01
9	Fruit and vegetable intake (Ch, T1)	5.65 (2.07)										.58 ***	.02	.01	.19 ***	.14 ***	−.01	−.02	< .01	.07 *	.05	.15 ***	−.03	.07 *
10	Fruit and vegetable intake (Ch, T2)	5.57 (1.65)											−.07 *	< .01	.10 **	.22 ***	−.01	< .01	< .01	.05	.05	.11 ***	−.05	.06
11	Energy‐dense food intake (P, T1)	3.43 (1.65)												.72 ***	.17 ***	.19 ***	.03	.04	.05	.04	−.19 ***	−.05	< .01	.08 **
12	Energy‐dense food intake (P, T2)	3.39 (1.40)													.17 ***	.27 ***	.01	.02	.04	.06	−.17 ***	−.04	−.05	.03
13	Energy‐dense food intake (Ch, T1)	4.55 (2.08)														.63 ***	.03	.04	06	.09 **	< .01	.20 ***	−.03	−.11 ***
14	Energy‐dense food intake (Ch, T2)	4.34 (1.63)															.03	.05	.03	.11 ***	−.01	.17 ***	−.01	−.08
15	BMI (P, T1)	24.57 (4.44)																.98 ***	.26 ***	.01	.12 ***	.01	−.19 ***	.01
16	BMI (P, T2)	24.55 (4.29)																	.26 ***	< .01	.13 ***	.01	−.19 ***	.01
17	BMI *z*‐score (Ch, T1)	0.37 (1.33)																		.21 ***	−.05	.06	.01	−.03
18	BMI *z*‐score (Ch, T2)	0.35 (1.01)																			−.04	.05	.04	.06
19	Age (P, T1)	36.75 (5.50)																				.20 ***	−.18 ***	< .01
20	Age (Ch, T1)	8.24 (1.41)																					−.06	< .01
21	Gender (P)																							.04
22	Gender (Ch)																							

Note

*** *p* < .001;** *p* < .01; * *p* < .05; T1 = Time 1, the baseline; T2 = Time 2, the 10‐month follow‐up; P = Parent; Ch = Child; BMI = body mass index.

### Indirect Effects of Parental Self‐Efficacy on Child Food Intake via Parental and Child Suppression (Model 1)

**FIGURE 1:**
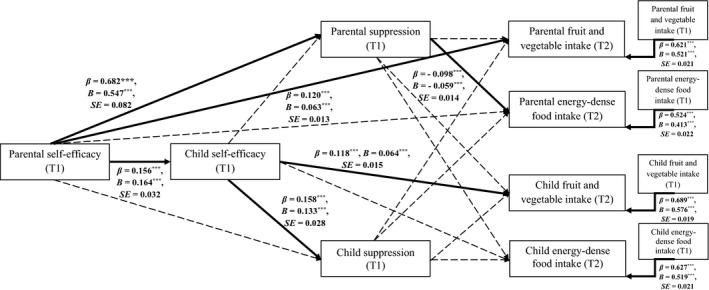
Direct associations for the hypothesised model: the direct effects of parental self‐efficacy on child food intake via parental and child suppression (*N* = 924 parent‐child dyads). *Note*: *** *p* < .001; ** *p* < .01; * *p* < .05. Only significant and unstandardised effect coefficients (*B*) are presented along bold arrows. Standard error (*SE*) is provided for unstandardised effect coefficients (*B*). Control variables and residuals of child and parental T1 mediators and dependent variables were assumed to covary. For clarity, the covariates are not displayed. The covariates include: parental and child gender, parental and child age (T1), parental BMI (T1 and T2), child BMI *z*‐score (T1 and T2). For values of all direct pathways, correlation, and covariance coefficients see Supplementary Table 1. T1 = Time 1, the baseline; T2 = Time 2, the 10‐month follow‐up.

The analyses conducted for the first hypothesised model, calculated for the total sample (*N* = 924 dyads; *N* = 1,848 individuals), indicated that the model‐data fit was acceptable, with *χ* ^2^ (118) = 386.204, *p* < .001, *χ* ^2^/*df* = 3.273, GFI = .962, NFI = .939, TLI = .930, CFI = .957, RMSEA = .050, 90% CI [.044, .055], SRMR = .050. The standardised and unstandardised path coefficients obtained for Model 1 are presented in Table 2. Overall, the variables in the hypothesised model explained 33.57 per cent of child fruit and vegetable intake (T2), and 39.56 per cent of child energy‐dense food intake (T2) (see Figure 1).

**TABLE 2:** The Standardised and Unstandardised Path Coefficients for the Hypothesised Models for the Indirect Effects between Parental Self‐Efficacy and Child Food Intake (*N* = 924 Parent‐Child Dyads)

*The model: assumed indirect pathways*	*Indirect effect*			
	*β*	*B*	*SE*	*95% CI*
*Model 1: The hypothesised model for the mediating effect of suppression*				
*Hypothesis 1* Self‐efficacy (P, T1) ➔ mediators (self‐efficacy [Ch, T1], suppression [P, T1], suppression [Ch, T1]) ➔ fruit and vegetable intake (Ch, T2)	0.036	0.020	0.011	< −0.001, 0.044
*Hypothesis 2* Self‐efficacy (P, T1) ➔ mediators (self‐efficacy [Ch, T1], suppression [P, T1], suppression [Ch, T1]) ➔ energy‐dense food intake (Ch, T2)	−0.010	−0.006	0.011	−0.029, 0.015
*Model 2: The hypothesised model for the mediating effect of distraction*				
*Hypothesis 3* Self‐efficacy (P, T1) ➔ mediators (self‐efficacy [Ch, T1], distraction [P, T1], distraction [Ch, T1]) ➔ fruit and vegetable intake (P, T2)	**0.023**	**0.021**	**0.009**	**0.006,** **0.043**
*Hypothesis 4* Self‐efficacy (P, T1) ➔ mediators (self‐efficacy [Ch, T1], distraction [P, T1], distraction [Ch, T1]) ➔ energy‐dense food intake (P, T2)	< 0.001	< −0.001	0.009	−0.019, 0.018

Note

Values of indirect effect coefficient (*B*) presented in bold are significant. CI = confidence intervals (bootstrap‐based, 10,000 repetitions); T1 = Time 1, the baseline; T2 = Time 2, the 10‐month follow‐up; P = Parent; Ch = Child.

The results of the path analysis for Hypothesis 1 and Hypothesis 2 (Table 2) showed that the associations between parental self‐efficacy (T1) and the two dependent variables, child fruit and vegetable intake (T2) and child energy‐dense food intake (T2), were not mediated by child self‐efficacy (T1) and parental and child suppression (T1). A significant indirect effect of parental self‐efficacy (T1) on child fruit and vegetable intake (T2) was found, with child self‐efficacy (T1) operating as the mediator, *β* = 0.019; *B* = 0.011, *p* < .001; *SE* = 0.003; 95% CI [0.006, 0.019]. Analyses performed to test possible indirect effects of parental self‐efficacy (T1) on parental food intake (T2) through parental and child suppression (T1) yielded non‐significant results.

Several direct associations between the variables included in Model 1 were found to be significant. High levels of self‐efficacy in parents (T1) were associated with high levels of the mediator parental suppression (T1). High levels of parental suppression (T1) predicted lower parental energy‐dense food intake in parents (T2). Furthermore, high levels of parental self‐efficacy (T1) were associated with high levels of child self‐efficacy (T1). High levels of child self‐efficacy (T1) predicted high fruit and vegetable intake among children (T2). All direct associations found for Model 1 are presented in Supplementary Table 1 and Figure 1.

### Indirect Effects of Parental Self‐Efficacy on Child Food Intake via Parental and Child Distraction (Model 2)

**FIGURE 2:**
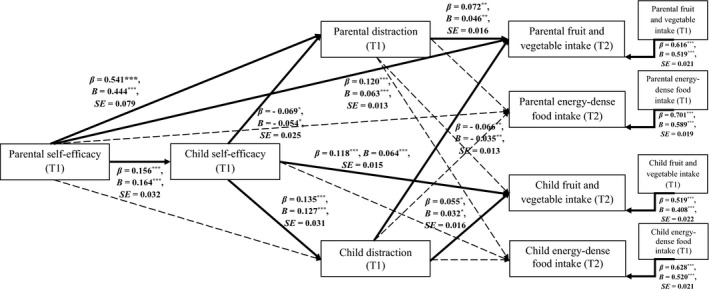
Direct associations for the hypothesised model: the direct effects of parental self‐efficacy on child food intake via parental and child distraction (*N* = 924 parent‐child dyads). *Note*: *** *p* < .001; ** *p* < .01; * *p* < .05. Only significant and unstandardised effect coefficients (*B*) are presented along bold arrows. Standard error (*SE*) is provided for unstandardised effect coefficients (*B*). Control variables and residuals of child and parental T1 mediators and dependent variables were assumed to covary. For clarity, the covariates are not displayed. The covariates include: parental and child gender, parental and child age (T1), parental BMI (T1 and T2), child BMI *z*‐score (T1 and T2). For values of all direct pathways, correlation, and covariance coefficients see Supplementary Table 1. T1 = Time 1, the baseline; T2 = Time 2, the 10‐month follow‐up.

In the case of the second hypothesised model calculated for the total sample (*N* = 924 dyads; *N* = 1,848 individuals), the model‐data fit was good, with *χ* ^2^ (118) = 432.231, *p *< .001, *χ* ^2^/*df* = 3.663, GFI = .958, NFI = .932, TLI = .950, CFI = .949, RMSEA = .054, 90% CI [.048, .059], SRMR = .054. The standardised and unstandardised path coefficients obtained for Model 2 are presented in Table 2. Overall, the variables in the hypothesised model explained 33.28 per cent of child fruit and vegetable intake (T2), and 39.64 per cent of child energy‐dense food intake (T2) (see Figure 2).

The results of the path analysis for Hypothesis 3 (Table 2) showed that the association between parental self‐efficacy (T1) and child fruit and vegetable intake (T2) was mediated by parental and child distraction (T1), as indicated by the significant indirect effect (*β* = 0.023; *B* = 0.021, *p* < .001; *SE* = 0.009; 95% CI [0.006, 0.043]). For Hypothesis 4, no significant indirect effect of parental self‐efficacy (T1) on child energy‐dense food intake (T2) was found. A significant indirect effect was found for parental self‐efficacy (T1) predicting child fruit and vegetable intake (T2) via child self‐efficacy (T1), *β* = 0.019; *B* = 0.011, *p* < .001; *SE* = 0.003; 95% CI [0.006, 0.019]. Analyses performed to test possible indirect effects of parental self‐efficacy (T1) on parental food intake (T2) through parental and child distraction (T1) yielded non‐significant results.

Several direct associations were found to be significant. High levels of self‐efficacy in parents (T1) predicted high parental fruit and vegetable intake (T2). Moreover, higher levels of self‐efficacy in parents (T1) were associated with high levels of parental distraction (T1). High levels of parental distraction (T1) predicted high parental fruit and vegetable intake (T2). Furthermore, high levels of parental self‐efficacy (T1) were associated with high levels of child self‐efficacy (T1). High levels of child self‐efficacy (T1) were associated with high child fruit and vegetable intake (T2) and were also associated with high levels of child distraction (T1). Child distraction (T1) predicted higher child fruit and vegetable intake (T2). Direct associations between the variables in Model 2 are presented in Supplementary Table 2 and Figure 2.

## DISCUSSION

This is the first prospective study to describe the interplay between self‐efficacy and self‐regulation indicators of child food intake among parent‐child dyads. Specifically, higher levels of parental self‐efficacy explained higher child fruit and vegetable intake through child self‐efficacy and child or parental distraction. In contrast, no indirect effects of parental self‐efficacy on child energy‐dense food intake were found, with child self‐efficacy, parental or child distraction assumed to mediate this relationship. We did not find indirect effects of parental self‐efficacy on child fruit and vegetable intake or child energy‐dense food intake, via child self‐efficacy, parental suppression, or child suppression.

High levels of parental self‐efficacy were found to be associated with higher levels of child self‐efficacy. This result is in line with social cognitive theory (Bandura, 1997) and socio‐ecological models (Davison & Birch, 2001; Townsend & Foster, 2011) that highlight the parental role in shaping children’s beliefs (such as self‐efficacy) and behaviors (such as self‐regulation strategies and food intake). Self‐efficacious children are more likely to use a self‐regulation strategy, distraction, which is congruent with previous findings (Anderson et al., 2007; Rothman et al., 2004; Schwarzer, 2008). High levels of child self‐efficacy and high levels of child distraction sequentially mediated the association between parental self‐efficacy and child fruit and vegetable intake. Similar findings were obtained in previous cross‐sectional research that confirmed bivariate associations between parental and child self‐efficacy as well as child self‐regulation with fruit and vegetable intake among children (Baranowski et al., 2010; Kokolaki et al., 2018; Norman et al., 2017; Parekh et al., 2017).

The results of our study provide further evidence for the need to broaden health behavior change models that are focused on individual factors (Hagger, 2016) within a family context, especially when child health behavior is taken into account. In line with socio‐ecological approaches (Davison & Birch, 2001), the broader models of health behavior change may claim that parental beliefs may explain child beliefs and, in consequence, child behavior.

The study has several limitations that need to be considered when interpreting the findings. Only self‐efficacy for fruit and vegetable intake was measured in the study. It would be optimal to measure self‐efficacy for energy‐dense food intake reduction as well as to test models including both types of parental and child self‐efficacy, and both types of parental and child self‐regulation (suppression and distraction). Yet, the associations between the variables in the model may result in multicollinearity, and thus two separate models were tested. In the present study these factors were not accounted for as mediators. Furthermore, participants were asked to recall the number of times they ate a portion of fruit and vegetables or energy‐dense food in the previous two weeks. Using such a measure is not ideal as it might be linked to retrospective bias or social desirability, which were not controlled for in the study. Future studies should consider using other methods, such as audio/video/photo/written records of meals, in‐depth dietary interviews, or food record software (Guan et al., 2019). Yet, the feasibility of using such measures of food intake in large samples is limited. Moreover, the study has not accounted for many other variables that may affect food intake, such as: socioeconomic variables (e.g. actual income; Li et al., 2019), emotional variables (e.g. food aversion; Zarychta et al., 2019), environmental variables (e.g. food accessibility, food scarcity; Luszczynska et al., 2013; van Rongen et al., 2019), or other key cognitions (e.g. self‐control; Hagger et al., 2019). Also, we did not screen parents or children for food allergies or food intolerances that may have contributed to a specific lower food intake. Future research could consider the inclusion of measures of these variables and using them as covariates. Furthermore, only one follow‐up measurement was applied, and the time interval between the baseline and the follow‐up measurement was relatively short to enable the study to be conducted during one school year. Future research should use at least two longer follow‐ups, with the independent variables, mediator variables, and dependent variables measured at different time points to enable the establishment of the temporal precedence of variables. Moreover, the results may have been affected by the sample characteristics in terms of the majority of parental participants being mothers. The moderating effect of gender could not be tested due to the small size of the father‐child sub‐sample, and despite controlling for both parent and child gender, the extent to which gender‐specific differences between study variables exist needs to be clarified in the future. Besides gender, we controlled for variables indicated previously as being significant confounds of food intake (such as baseline food intake, age, BMI, and BMI *z*‐score), which can explain the low beta coefficients obtained in the study. Moreover, any generalisation to ethnically diverse populations should be made cautiously since all participants enrolled in the study were Caucasian.

The findings of the study are particularly important since food intake is assumed to be habitual and difficult to modify (Churchill et al., 2018). In this context, the recognition of modifiable predictors and mediators of food intake is of key significance in creating effective health promotion programs. Importantly, the results of the study indicate that for child fruit and vegetable intake, it is the interplay between parental self‐efficacy, child self‐efficacy, and distraction strategies that is crucial. Therefore, it can be concluded that programs aiming at changing fruit and vegetable intake among 5–11‐year‐old children should target both enhancing self‐efficacy and distraction strategies, and include both parents and children.

## Funding

The contribution of Anna Banik and Ewa Kulis was supported by grant 2014/15/B/HS06/00923 awarded by the National Science Centre, Poland. Open access of this article was financed by the Ministry of Science and Higher Education in Poland under the 2019‐2022 program “Regional Initiative of Excellence”, project number 012/RID/2018/19.

## Conflict of Interest

The authors declare that they have no conflict of interest.

## Supporting information


**Table S1.** Standardized and Unstandardized Path Coefficients and Covariance Coefficients for the Hypothesized Model: The Direct Effects of Parental Self‐Efficacy on Child Food Intake via Parental and Child Suppression (N = 924 Parent‐Child Dyads).
**Table S2.** Standardized and Unstandardized Path Coefficients and Covariance Coefficients for the Hypothesized Model: The Direct Effects of Parental Self‐Efficacy on Child Food Intake via Parental and Child Distraction (N = 924 parent‐child dyads).
