# Supplementary material for: Parental and Child Self‐Efficacy Explaining Food Intake through Self‐Regulation: A Dyadic Prospective Study
Source: Appl Psychol Health Well Being. 2020 Sep 13;13(1):174–94. doi: 10.1111/aphw.12225 (PMC7891348; doi:10.1111/aphw.12225)
Supplement: Supplementary file 1 — Table S1. Standardized and Unstandardized Path Coefficients and Covariance Coefficients for the Hypothesized Model: The Direct Effects of Parental Self‐Efficacy on Child Food Intake via Parental and Child Suppression (N = 924 Parent‐Child Dyads). Table S2. Standardized and Unstandardized Path Coefficients and Covariance Coefficients for the Hypothesized Model: The Direct Effects of Parental Self‐Efficacy on Child Food Intake via Parental and Child Distraction (N = 924 parent‐child dyads). [file APHW-13-174-s001.docx]

**Parental and Child Self-Efficacy Explaining Food Intake Through Self-Regulation: A Dyadic Prospective Study**

**Supplement 1**

Supplement 1 includes the results of the additional analyses. Standardized and unstandardized direct effect coefficients and covariance coefficients for the hypothesized models (*N* = 924 parent-child dyads) are presented in the Supplementary Table 1 (for suppression tested as a mediator) and Supplementary Table 2 (for distraction tested as a mediator).

**Table 1**

*Standardized and Unstandardized Path Coefficients and Covariance Coefficients for the Hypothesized Model: The Direct Effects of Parental Self-Efficacy on Child Food Intake via Parental and Child Suppression (N = 924 Parent-Child Dyads)*

| Path coefficients/covariance coefficients | *β* | *B* | *SE* | *p*-value |
| --- | --- | --- | --- | --- |
| **Predictors of study variables** |  |  |  |  |
| Self-efficacy (P, T1) 🡪 Self-efficacy (Ch, T1) | **0.156** | **0.164** | **0.032** | **< .001** |
| Self-efficacy (P, T1) 🡪 Suppression (P, T1) | **0.682** | **0.547** | **0.082** | **< .001** |
| Self-efficacy (P, T1) 🡪 Suppression (Ch, T1) | 0.002 | 0.002 | 0.029 | .945 |
| Self-efficacy (P, T1) 🡪 Fruit and vegetable intake (P, T2) | **0.120** | **0.063** | **0.013** | **< .001** |
| Self-efficacy (P, T1) 🡪 Energy-dense food intake (P, T2) | - 0.019 | - 0.009 | 0.011 | .409 |
| Self-efficacy (Ch, T1) 🡪 Suppression (P, T1) | 0.002 | 0.002 | 0.025 | .948 |
| Self-efficacy (Ch, T1) 🡪 Suppression (Ch, T1) | **0.158** | **0.133** | **0.028** | **< .001** |
| Self-efficacy (Ch, T1) 🡪 Fruit and vegetable intake (Ch, T2) | **0.118** | **0.064** | **0.015** | **< .001** |
| Self-efficacy (Ch, T1) 🡪 Energy-dense food intake (Ch, T2) | - 0.020 | - 0.011 | 0.015 | .437 |
| Suppression (P, T1) 🡪 Fruit and vegetable intake (Ch, T2) | 0.024 | 0.017 | 0.019 | .376 |
| Suppression (P, T1) 🡪 Energy-dense food intake (Ch, T2) | - 0.010 | - 0.008 | 0.019 | .685 |
| Suppression (P, T1) 🡪 Fruit and vegetable intake (P, T2) | 0.020 | 0.013 | 0.016 | .416 |
| Suppression (P, T1) 🡪 Energy-dense food intake (P, T2) | **- 0.098** | **- 0.059** | **0.014** | **< .001** |
| Suppression (Ch, T1) 🡪 Fruit and vegetable intake (Ch, T2) | 0.039 | 0.025 | 0.018 | .150 |
| Suppression (Ch, T1) 🡪 Energy-dense food intake (Ch, T2) | - 0.007 | - 0.005 | 0.018 | .793 |
| Suppression (Ch, T1) 🡪 Energy-dense food intake (P, T2) | - 0.009 | - 0.005 | 0.013 | .706 |
| Suppression (Ch, T1) 🡪 Fruit and vegetable intake (P, T2) | - 0.004 | - 0.002 | 0.014 | .869 |
| Fruit and vegetable intake (P, T1) 🡪 Fruit and vegetable intake (P, T2) | **0.621** | **0.521** | **0.021** | **< .001** |
| Fruit and vegetable intake (Ch, T1) 🡪 Fruit and vegetable intake (Ch, T2) | **0.524** | **0.413** | **0.022** | **< .001** |
| Energy-dense food intake (P, T1) 🡪 Energy-dense food intake (P, T2) | **0.689** | **0.576** | **0.019** | **< .001** |
| Energy-dense food intake (Ch, T1) 🡪 Energy-dense food intake (Ch, T2) | **0.627** | **0.519** | **0.021** | **< .001** |
| **Covariances** |  |  |  |  |
| Self-efficacy (P, T1) 🡨🡪 Fruit and vegetable intake (P, T1) | **0.308** | **1.563** | **0.168** | **< .001** |
| Self-efficacy (P, T1) 🡨🡪 Energy-dense food intake (P, T1) | **- 0.187** | **- 0.876** | **0.139** | **< .001** |
| Self-efficacy (P, T1) 🡨🡪 Suppression (P, T1) | **- 0.496** | **- 3.701** | **0.633** | **< .001** |
| Suppression (Ch, T1) 🡨🡪 Suppression (P, T1) | **0.096** | **0.624** | **0.183** | **< .001** |
| Fruit and vegetable intake (P, T1) 🡨🡪 BMI (P, T1) | **- 0.086** | **- 0.678** | **0.229** | **.003** |
| Fruit and vegetable intake (P, T1) 🡨🡪 BMI (P, T2) | **- 0.083** | **- 0.629** | **0.221** | **.004** |
| Fruit and vegetable intake (P, T1) 🡨🡪 Age (P, T1) | - 0.029 | - 0.278 | 0.293 | .343 |
| Fruit and vegetable intake (P, T1) 🡨🡪 Fruit and vegetable intake (Ch, T1) | **0.104** | **0.380** | **0.105** | **< .001** |
| Fruit and vegetable intake (P, T1) 🡨🡪 Energy-dense food intake (Ch, T1) | - 0.003 | - 0.010 | 0.110 | .930 |
| Fruit and vegetable intake (P, T2) 🡨🡪 Fruit and vegetable intake (Ch, T2) | **0.177** | **0.258** | **0.048** | **< .001** |
| Fruit and vegetable intake (Ch, T1) 🡨🡪 Energy-dense food intake (Ch, T1) | **0.185** | **0.793** | **0.143** | **< .001** |
| Fruit and vegetable intake (Ch, T1) 🡨🡪 BMI z-score (Ch, T1) | - 0.001 | - 0.002 | 0.080 | .986 |
| Fruit and vegetable intake (Ch, T1) 🡨🡪 BMI z-score (Ch, T2) | 0.056 | 0.116 | 0.064 | .068 |
| Fruit and vegetable intake (Ch, T1) 🡨🡪 Age (Ch, T1) | **0.090** | **0.261** | **0.088** | **.003** |
| Fruit and vegetable intake (Ch, T1) 🡨🡪 Self-efficacy (Ch, T1) | **0.339** | **2.065** | **0.209** | **< .001** |
| Energy-dense food intake (P, T1) 🡨🡪 BMI (P, T1) | 0.009 | 0.065 | 0.219 | .766 |
| Energy-dense food intake (P, T1) 🡨🡪 BMI (P, T2) | 0.020 | 0.143 | 0.212 | .500 |
| Energy-dense food intake (P, T1) 🡨🡪 Fruit and vegetable intake (P, T1) | 0.008 | 0.025 | 0.096 | .797 |
| Energy-dense food intake (P, T1) 🡨🡪 Age (P, T1) | **- 0.168** | **- 1.519** | **0.285** | **< .001** |
| Energy-dense food intake (P, T1) 🡨🡪 Fruit and vegetable intake (Ch, T1) | **0.077** | **0.262** | **0.100** | **.009** |
| Energy-dense food intake (P, T1) 🡨🡪 Energy-dense food intake (Ch, T1) | **0.158** | **0.542** | **0.107** | **< .001** |
| Energy-dense food intake (P, T2) 🡨🡪 Fruit and vegetable intake (P, T2) | **0.108** | **0.116** | **0.035** | **< .001** |
| Energy-dense food intake (P, T2) 🡨🡪 Energy-dense food intake (Ch, T2) | **0.157** | **0.204** | **0.042** | **< .001** |
| Energy-dense food intake (Ch, T1) 🡨🡪 BMI z-score (Ch, T1) | 0.044 | 0.121 | 0.086 | .163 |
| Energy-dense food intake (Ch, T1) 🡨🡪 BMI z-score (Ch, T2) | **0.096** | **0.203** | **0.068** | **.003** |
| Energy-dense food intake (Ch, T1) 🡨🡪 Age (Ch, T1) | **0.221** | **0.650** | **0.096** | **< .001** |
| Energy-dense food intake (Ch, T1) 🡨🡪 Self-efficacy (Ch, T1) | **- 0.070** | **- 0.433** | **0.195** | **.027** |
| Energy-dense food intake (Ch, T2) 🡨🡪 Fruit and vegetable intake (Ch, T2) | **0.200** | **0.355** | **0.058** | **< .001** |
| BMI (P, T1) 🡨🡪 BMI (P, T2) | **0.978** | **18.516** | **0.868** | **< .001** |
| BMI (P, T1) 🡨🡪 BMI z-score (Ch, T2) | 0.019 | 0.085 | 0.142 | .549 |
| BMI (P, T1) 🡨🡪 BMI z-score (Ch, T1) | **0.269** | **1.585** | **0.195** | **< .001** |
| BMI (P, T1) 🡨🡪 Fruit and vegetable intake (P, T2) | 0.028 | 0.139 | 0.148 | .350 |
| BMI (P, T1) 🡨🡪 Energy-dense food intake (P, T2) | - 0.022 | - 0.095 | 0.131 | .465 |
| BMI (P, T2) 🡨🡪 BMI z-score (Ch, T2) | 0.014 | 0.059 | 0.137 | .670 |
| BMI (P, T2) 🡨🡪 BMI z-score (Ch, T1) | **0.272** | **1.542** | **0.189** | **< .001** |
| BMI (P, T2) 🡨🡪 Fruit and vegetable intake (P, T2) | 0.014 | 0.066 | 0.143 | .645 |
| BMI (P, T2) 🡨🡪 Energy-dense food intake (P, T2) | - 0.010 | - 0.040 | 0.126 | .752 |
| BMI z-score (Ch, T1) 🡨🡪 BMI z-score (Ch, T2) | **0.211** | **0.284** | **0.045** | **< .001** |
| BMI z-score (Ch, T1) 🡨🡪 Fruit and vegetable intake (Ch, T2) | - 0.006 | - 0.010 | 0.055 | .855 |
| BMI z-score (Ch, T2) 🡨🡪 Fruit and vegetable intake (Ch, T2) | 0.008 | 0.011 | 0.043 | .803 |
| BMI z-score (Ch, T1) 🡨🡪 Energy-dense food intake (Ch, T2) | - 0.026 | - 0.046 | 0.055 | .403 |
| BMI z-score (Ch, T2) 🡨🡪 Energy-dense food intake (Ch, T2) | **0.064** | **0.087** | **0.044** | **.046** |
| Gender (P, T1) 🡨🡪 BMI (P, T1) | **- 0.171** | **- 0.237** | **0.044** | **< .001** |
| Gender (P, T1) 🡨🡪 BMI (P, T2) | **- 0.160** | **- 0.215** | **0.042** | **< .001** |
| Gender (Ch, T1) 🡨🡪 BMI z-score (Ch, T2) | **0.072** | **0.036** | **0.017** | **.028** |
| Gender (Ch, T1) 🡨🡪 BMI z-score (Ch, T1) | - 0.033 | - 0.022 | 0.021 | .290 |
| Age (P, T1) 🡨🡪 BMI (P, T2) | **0.119** | **2.791** | **0.724** | **< .001** |
| Age (P, T1) 🡨🡪 BMI (P, T1) | **0.105** | **2.546** | **0.748** | **< .001** |
| Age (Ch, T1) 🡨🡪 Age (P, T1) | **0.194** | **1.505** | **0.247** | **< .001** |
| Age (Ch, T1) 🡨🡪 BMI z-score (Ch, T2) | 0.054 | 0.078 | 0.046 | .091 |
| Age (Ch, T1) 🡨🡪 BMI z-score (Ch, T1) | **0.070** | **0.131** | **0.058** | **.025** |

*Note.* T1 = Time 1, the baseline; T2 = Time 2, the 10-month follow-up; P = Parent; Ch = Child; BMI = body mass index. Significant coefficients are marked in bold.

**Table 2**

*Standardized and Unstandardized Path Coefficients and Covariance Coefficients for the Hypothesized Model: The Direct Effects of Parental Self-Efficacy on Child Food Intake via Parental and Child Distraction (N = 924 parent-child dyads)*

| Path coefficients/covariance coefficients | *β* | *B* | *SE* | *p*-value |
| --- | --- | --- | --- | --- |
| **Predictors of study variables** |  |  |  |  |
| Self-efficacy (P, T1) 🡪 Self-efficacy (Ch, T1) | **0.156** | **0.164** | **0.032** | **< .001** |
| Self-efficacy (P, T1) 🡪 Distraction (P, T1) | **0.541** | **0.444** | **0.079** | **< .001** |
| Self-efficacy (P, T1) 🡪 Distraction (Ch, T1) | 0.032 | 0.032 | 0.033 | .332 |
| Self-efficacy (P, T1) 🡪 Fruit and vegetable intake (P, T2) | **0.120** | **0.063** | **0.013** | **< .001** |
| Self-efficacy (P, T1) 🡪 Energy-dense food intake (P, T2) | - 0.027 | - 0.013 | 0.011 | .252 |
| Self-efficacy (Ch, T1) 🡪 Distraction (P, T1) | **- 0.069** | **- 0.054** | **0.025** | **.034** |
| Self-efficacy (Ch, T1) 🡪 Distraction (Ch, T1) | **0.135** | **0.127** | **0.031** | **< .001** |
| Self-efficacy (Ch, T1) 🡪 Fruit and vegetable intake (Ch, T2) | **0.119** | **0.064** | **0.015** | **< .001** |
| Self-efficacy (Ch, T1) 🡪 Energy-dense food intake (Ch, T2) | - 0.019 | - 0.011 | 0.015 | .453 |
| Distraction (P, T1) 🡪 Fruit and vegetable intake (Ch, T2) | 0.036 | 0.025 | 0.019 | .181 |
| Distraction (P, T1) 🡪 Energy-dense food intake (Ch, T2) | 0.006 | 0.004 | 0.019 | .831 |
| Distraction (P, T1) 🡪 Fruit and vegetable intake (P, T2) | **0.072** | **0.046** | **0.016** | **.004** |
| Distraction (P, T1) 🡪 Energy-dense food intake (P, T2) | - 0.037 | - 0.022 | 0.014 | .119 |
| Distraction (Ch, T1) 🡪 Fruit and vegetable intake (Ch, T2) | **0.055** | **0.032** | **0.016** | **.043** |
| Distraction (Ch, T1) 🡪 Energy-dense food intake (Ch, T2) | - 0.002 | - 0.001 | 0.016 | .950 |
| Distraction (Ch, T1) 🡪 Energy-dense food intake (P, T2) | 0.031 | 0.015 | 0.011 | .179 |
| Distraction (Ch, T1) 🡪 Fruit and vegetable intake (P, T2) | **- 0.066** | **- 0.035** | **0.013** | **.006** |
| Fruit and vegetable intake (P, T1) 🡪 Fruit and vegetable intake (P, T2) | **0.616** | **0.519** | **0.021** | **< .001** |
| Fruit and vegetable intake (Ch, T1) 🡪 Fruit and vegetable intake (Ch, T2) | **0.519** | **0.408** | **0.022** | **< .001** |
| Energy-dense food intake (P, T1) 🡪 Energy-dense food intake (P, T2) | **0.701** | **0.589** | **0.019** | **< .001** |
| Energy-dense food intake (Ch, T1) 🡪 Energy-dense food intake (Ch, T2) | **0.628** | **0.520** | **0.021** | **< .001** |
| **Covariances** |  |  |  |  |
|  |  |  |  |  |
| Self-efficacy (P, T1) 🡨🡪 Fruit and vegetable intake (P, T1) | **0.323** | **1.637** | **0.171** | **< .001** |
| Self-efficacy (P, T1) 🡨🡪 Energy-dense food intake (P, T1) | **- 0.167** | **- 0.785** | **0.143** | **< .001** |
| Self-efficacy (P, T1) 🡨🡪 Distraction (P, T1) | **- 0.394** | **- 2.836** | **0.601** | **< .001** |
| Distraction (Ch, T1) 🡨🡪 Distraction (P, T1) | **0.105** | **0.740** | **0.211** | **< .001** |
| Fruit and vegetable intake (P, T1) 🡨🡪 Age (P, T1) | - 0.030 | - 0.294 | 0.293 | .316 |
| Fruit and vegetable intake (P, T1) 🡨🡪 Fruit and vegetable intake (Ch, T1) | **0.102** | **0.372** | **0.106** | **< .001** |
| Fruit and vegetable intake (P, T1) 🡨🡪 Energy-dense food intake (Ch, T1) | - 0.002 | - 0.006 | 0.110 | .959 |
| Fruit and vegetable intake (P, T1) 🡨🡪 BMI (P, T1) | **- 0.091** | **- 0.715** | **0.230** | **.002** |
| Fruit and vegetable intake (P, T1) 🡨🡪 BMI (P, T2) | **- 0.088** | **- 0.667** | **0.222** | **.003** |
| Fruit and vegetable intake (P, T2) 🡨🡪 Energy-dense food intake (P, T2) | **0.114** | **0.121** | **0.034** | **< .001** |
| Fruit and vegetable intake (P, T2) 🡨🡪 Energy-dense food intake (Ch, T2) | **0.156** | **0.204** | **0.042** | **< .001** |
| Fruit and vegetable intake (Ch, T1) 🡨🡪 Energy-dense food intake (Ch, T1) | **0.185** | **0.792** | **0.143** | **< .001** |
| Fruit and vegetable intake (Ch, T1) 🡨🡪 BMI z-score (Ch, T1) | - 0.002 | - 0.007 | 0.081 | .993 |
| Fruit and vegetable intake (Ch, T1) 🡨🡪 BMI z-score (Ch, T2) | 0.056 | 0.116 | 0.064 | .068 |
| Fruit and vegetable intake (Ch, T1) 🡨🡪 Age (Ch, T1) | **0.089** | **0.260** | **0.088** | **.003** |
| Fruit and vegetable intake (Ch, T1) 🡨🡪 Self-efficacy (Ch, T1) | **0.339** | **2.067** | **0.209** | **< .001** |
| Energy-dense food intake (P, T1) 🡨🡪 BMI (P, T1) | 0.011 | 0.080 | 0.221 | .717 |
| Energy-dense food intake (P, T1) 🡨🡪 BMI (P, T2) | 0.022 | 0.157 | 0.213 | .461 |
| Energy-dense food intake (P, T1) 🡨🡪 Fruit and vegetable intake (P, T1) | 0.008 | 0.024 | 0.096 | .804 |
| Energy-dense food intake (P, T1) 🡨🡪 Age (P, T1) | **- 0.169** | **- 1.529** | **0.287** | **< .001** |
| Energy-dense food intake (P, T1) 🡨🡪 Fruit and vegetable intake (Ch, T1) | **0.075** | **0.254** | **0.101** | **.012** |
| Energy-dense food intake (P, T1) 🡨🡪 Energy-dense food intake (Ch, T1) | **0.158** | **0.544** | **0.107** | **< .001** |
| Energy-dense food intake (P, T2) 🡨🡪 Fruit and vegetable intake (Ch, T2) | **0.181** | **0.262** | **0.047** | **< .001** |
| Energy-dense food intake (Ch, T1) 🡨🡪 BMI z-score (Ch, T1) | 0.044 | 0.122 | 0.086 | .159 |
| Energy-dense food intake (Ch, T1) 🡨🡪 BMI z-score (Ch, T2) | **0.097** | **0.204** | **0.068** | **.003** |
| Energy-dense food intake (Ch, T1) 🡨🡪 Age (Ch, T1) | **0.221** | **0.651** | **0.096** | **< .001** |
| Energy-dense food intake (Ch, T1) 🡨🡪 Self-efficacy (Ch, T1) | **- 0.070** | **- 0.434** | **0.195** | **.026** |
| Energy-dense food intake (Ch, T2) 🡨🡪 Fruit and vegetable intake (Ch, T2) | **0.200** | **0.354** | **0.058** | **< .001** |
| BMI (P, T1) 🡨🡪 BMI (P, T2) | **0.978** | **18.505** | **0.867** | **< .001** |
| BMI (P, T1) 🡨🡪 BMI z-score (Ch, T2) | 0.020 | 0.087 | 0.142 | .540 |
| BMI (P, T1) 🡨🡪 BMI z-score (Ch, T1) | **0.269** | **1.581** | **0.195** | **< .001** |
| BMI (P, T1) 🡨🡪 Energy-dense food intake (P, T2) | 0.022 | 0.105 | 0.147 | .475 |
| BMI (P, T1) 🡨🡪 Fruit and vegetable intake (P, T2) | - 0.028 | - 0.122 | 0.132 | .353 |
| BMI (P, T2) 🡨🡪 BMI z-score (Ch, T2) | 0.014 | 0.061 | 0.137 | .656 |
| BMI (P, T2) 🡨🡪 BMI z-score (Ch, T1) | **0.271** | **1.540** | **0.188** | **< .001** |
| BMI (P, T2) 🡨🡪 Energy-dense food intake (P, T2) | 0.008 | 0.037 | 0.142 | .796 |
| BMI (P, T2) 🡨🡪 Fruit and vegetable intake (P, T2) | - 0.016 | - 0.067 | 0.127 | .598 |
| BMI z-score (Ch, T1) 🡨🡪 BMI z-score (Ch, T2) | **0.211** | **0.284** | **0.045** | **< .001** |
| BMI z-score (Ch, T1) 🡨🡪 Fruit and vegetable intake (Ch, T2) | - 0.013 | - 0.023 | 0.054 | .671 |
| BMI z-score (Ch, T1) 🡨🡪 Energy-dense food intake (Ch, T2) | - 0.026 | - 0.046 | 0.055 | .407 |
| BMI z-score (Ch, T2) 🡨🡪 Fruit and vegetable intake (Ch, T2) | 0.004 | 0.005 | 0.043 | .901 |
| BMI z-score (Ch, T2) 🡨🡪 Energy-dense food intake (Ch, T2) | **0.065** | **0.088** | **0.044** | **.045** |
| Gender (P, T1) 🡨🡪 BMI (P, T1) | **- 0.169** | **- 0.235** | **0.044** | **< .001** |
| Gender (P, T1) 🡨🡪 BMI (P, T2) | **- 0.159** | **- 0.213** | **0.042** | **< .001** |
| Gender (Ch, T1) 🡨🡪 BMI z-score (Ch, T2) | **0.072** | **0.036** | **0.017** | **.028** |
| Gender (Ch, T1) 🡨🡪 BMI z-score (Ch, T1) | - 0.033 | - 0.022 | 0.021 | .293 |
| Age (P, T1) 🡨🡪 BMI (P, T2) | **0.119** | **2.794** | **0.724** | **< .001** |
| Age (P, T1) 🡨🡪 BMI (P, T1) | **0.105** | **2.549** | **0.748** | **< .001** |
| Age (Ch, T1) 🡨🡪 Age (P, T1) | **0.194** | **1.505** | **0.247** | **< .001** |
| Age (Ch, T1) 🡨🡪 BMI z-score (Ch, T2) | 0.054 | 0.078 | 0.046 | .092 |
| Age (Ch, T1) 🡨🡪 BMI z-score (Ch, T1) | **0.070** | **0.131** | **0.058** | **.026** |

*Note.* T1 = Time 1, the baseline; T2 = Time 2, the 10-month follow-up; P = Parent; Ch = Child; BMI = body mass index. Significant coefficients are marked in bold.
